# Intraprocedural versus next day transthoracic echocardiography following minimalist transfemoral TAVI

**DOI:** 10.1186/s44156-023-00025-w

**Published:** 2023-09-07

**Authors:** Panagiotis Savvoulidis, M. Adnan Nadir, William E. Moody, Richard Steeds, Peter F. Ludman, Joseph R. Bradley, Aldrin Singh, Ewa Lawton, Sagar N. Doshi

**Affiliations:** 1 Department of Cardiology, Queen Elizabeth Hospital Birmingham, Mindelsohn Way, Edgbaston, Birmingham, B15 2WB UK; 2 Institute for Cardiovascular Sciences, College of Medical and Dental Sciences, University of Birmingham, Edgbaston, Birmingham, B15 2TT UK

**Keywords:** Transcatheter aortic valve implantation, Echocardiography guidance, Complications, Same day ECHO, Next day ECHO

## Abstract

**Background:**

Routine pre-discharge echocardiography (ECHO) is recommended post transcatheter aortic valve implantation (TAVI) as a baseline for future comparison. However, there is no clear guidance on the optimal timing of this study.

**Aim:**

The purpose of this retrospective study was to investigate the safety and work-force efficiency of intraprocedural same-day ECHO versus next-day ECHO, following transfemoral TAVI.

**Methods and results:**

In this retrospective study 100 consecutive patients who underwent intraprocedural ECHO only were compared with 100 consecutive patients undergoing both intraprocedural and routine next-day ECHO following elective transfemoral TAVI. All patients received the Sapien 3/Ultra transcatheter heart valve and were treated with a minimalist procedure with conscious sedation. The composite of in-hospital mortality, urgent ECHO and new tamponade after leaving the cath lab and before discharge was not different between the two groups (4 vs. 4%, P = 1). There was no paravalvular leak more than mild in any of the cases. Length of stay was similar (1 day).

**Conclusions:**

Intraprocedural post-TAVI ECHO appears as safe as next day pre-discharge ECHO and obviates the need for a routine next day study, thereby reducing burden on echocardiography services and allows better utilisation of resources.

## Introduction

The favourable results of transcatheter aortic valve implantation (TAVI) compared with open heart surgery in multiple randomized trials incorporating patients with severe symptomatic aortic stenosis across the entire risk spectrum have culminated in a Class I recommendation for TAVI in patients older than 75 years old or those at high surgical risk in the most recent European Society of Cardiology/European Association for Cardiothoracic Surgery guidelines. [1]

As the number of TAVI procedures is expected to significantly grow, with the broadening of indications and an increasingly ageing population, it is important that all refinements that improve efficiency and reduce cost, whilst retaining high standards of safety, should be adopted [2, 3]. Implementation of such optimization programs reduce the burden on hospital resources thereby minimizing costs and allow a more effective distribution of workforce. Echocardiographers are a precious and limited resource and a recent survey by the British Society of Echocardiography, commissioned to understand the current echocardiographer workforce in the UK and the issues it is facing, found that 53% of respondents to a survey (50/95) had reported failure to appoint to vacant echo-cardiographer posts following advertisement, suggesting a significant shortage of trained echocardiographers. [4]

Echocardiography (ECHO) post-TAVI is recommended as a baseline for future comparison [5]. However, there are no recommendations as to when this baseline study should be undertaken although a pre-discharge study would seem prudent and convenient [6, 7]. We recently published an intraprocedural transthoracic ECHO protocol to support minimalist TAVI procedures that recommended echocardiographic assessment at 3 distinct timepoints in the procedure (pre-TAVI, immediate post-TAVI and pre-cath lab exit) with the intention of focusing and streamlining echocardiographic examination to improve the efficiency and safety of the procedure [8]. In the protocol we further reasoned that the pre-cath lab exit study may obviate the need for a further, routine, next day or pre-discharge ECHO with the benefit of reducing overall cost and in particular reducing the pressure on echocardiography services. Intraprocedural ECHO may also facilitate same-day discharge in selected individuals. [9, 10]

In this retrospective study we aimed to investigate the safety and work-force efficiency of intraprocedural only versus additional next-day, pre-discharge ECHO following TAVI.

## Methods

### Study population

Between January 2019 and December 2020, we identified consecutive, elective patients who had transfemoral TAVI at our institution with the Edwards Sapien 3 or Sapien 3 Ultra transcatheter heart valve (THV) (Edwards Lifesciences, Irvine, California). This time frame was selected as in January 2020 our department switched from a policy of intraprocedural ECHO plus routine, next-day pre-discharge ECHO to intraprocedural ECHO only. 100 consecutive patients who underwent intraprocedural ECHO and a routine next day ECHO, before the policy change, were compared with the first 100 consecutive patients undergoing an intraprocedural ECHO only after this date.

### Methods

All patients were discussed by our Heart Team and were deemed suitable for trans-femoral TAVI with the balloon-expandable Edwards Sapien 3/Sapien 3 Ultra THV. All patients provided written informed consent for this procedure. As this was a retrospective study using a standard technique at our institution, ethical approval was not required by our Institutional Review Board and no special consent was required. All patients had a dedicated ECG-gated CT aortogram for vascular access selection and valve sizing purposes. All patients were admitted the evening prior to the TAVI. All procedures were undertaken with conscious sedation via transfemoral access and with continuous invasive monitoring. ECHO was undertaken by experienced sonographers using cardiac ECHO machines including the CX50 (Philips, Amsterdam, Netherlands), the HD11XE (Philips, Amsterdam, Netherlands). Measurements were undertaken as per the British Society of Echocardiography mandates [11]. More precisely, the aortic valve area calculation was based on the standard continuity Equation [12]. The details for the protocol for intraprocedural ECHO during transfemoral minimalist TAVI have been described previously by our group [8]. In brief, ECHO was undertaken immediately before the TAVI procedure, while the patient was on the cath lab table to identify appropriate acoustic windows and capture baseline anatomical features. This enabled slight changes in body posture and permitted identification of the best possible acoustic windows. At the conclusion of the procedure, the same body posture was used to undertake a comprehensive ECHO study. ECHO was repeated immediately after the deployment of the THV for safety reasons: to identify new pericardial collections and changes in left ventricular function. An assessment of paravalvular leak was also undertaken to guide further balloon dilatation. At the conclusion of the procedure, before cath lab exit, a comprehensive ECHO was repeated to acquire transvalvular gradient and measurements (for future reference), evaluation of residual paravalvular leak, to exclude new pericardial effusion collection, assess left ventricular function and mitral regurgitation. In the first cohort, in addition to the intraprocedural ECHO, a further routine ECHO was undertaken the following morning or prior to discharge for capture of baseline parameters. In the second cohort a routine next day or pre-discharge ECHO was not performed and the pre cath lab exit ECHO was used as the baseline study. In both patient groups the requirement for an emergent, unplanned ECHO in the case of a clinical change after leaving the cath lab and before discharge was recorded.

### Endpoints

The primary endpoint was a composite of in-hospital mortality, need for urgent ECHO (unplanned urgent ECHO to assist in the differential diagnosis of hypotension, chest pain, hypoxemia) before discharge and new tamponade detected after leaving the cath lab. Secondary endpoints were total rates of cardiac tamponade, severity of para-valvular leak, length of stay, 30-day mortality and 30-day cardiac readmissions including heart failure, acute coronary syndromes, arrhythmias.

### Statistical analysis

Continuous data are presented as median and interquartile range and were compared between groups using the Mann–Whitney test. Categorical data are presented as numbers (N) and frequencies (percentages) and were compared using the Fisher exact test. All statistical tests were 2-sided and P values < 0.05 were considered statistically significant. Statistical analyses were performed using R version 4.1.1 (R Foundation for Statistical Computing).

## Results

**Fig. 1:**
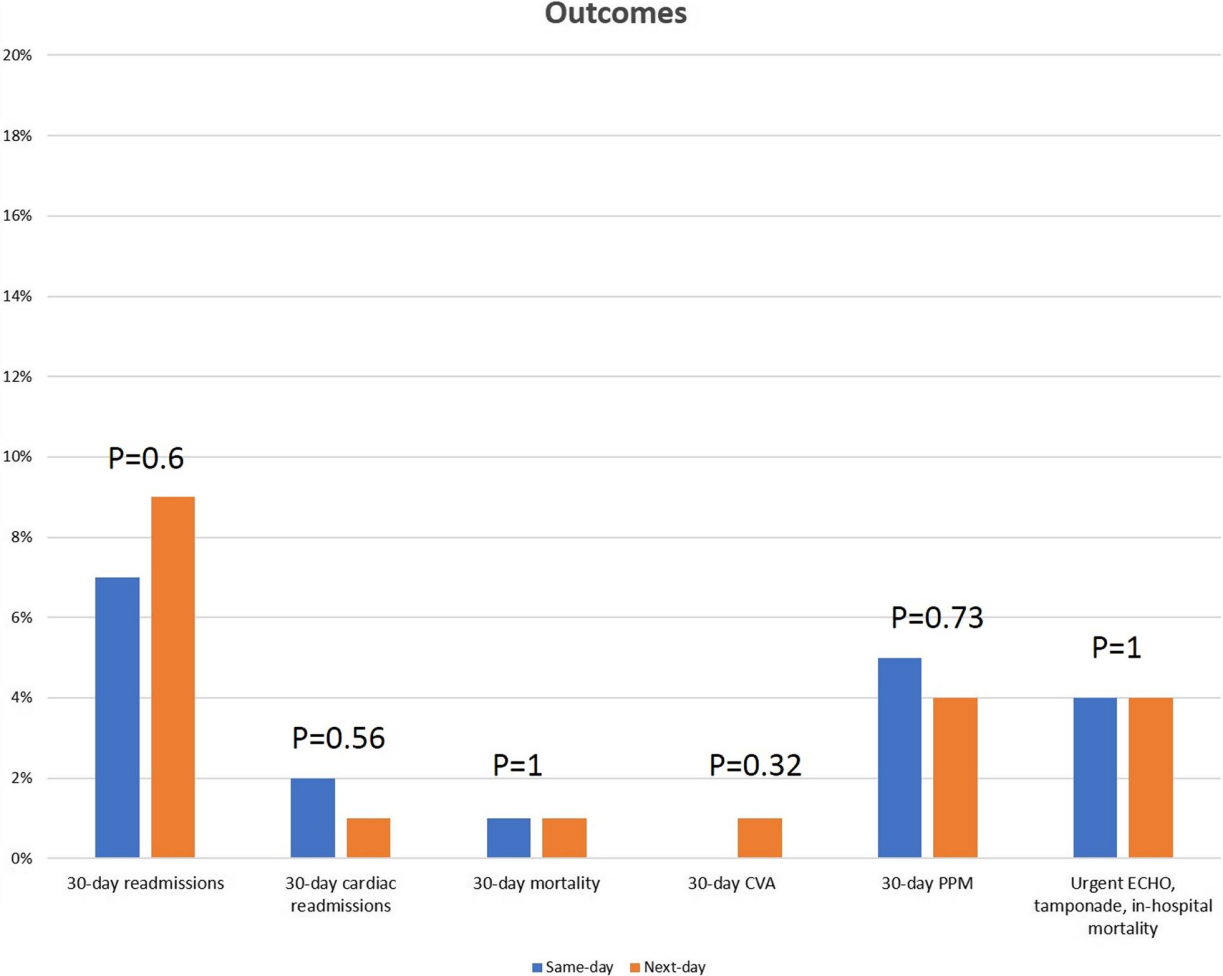
Outcomes and comparison between the same-day and next-day ECHO cohorts

The total cohort comprised of 200 patients; 100 undergoing intraprocedural and next day transthoracic ECHO and 100 undergoing intraprocedural transthoracic ECHO only. Baseline demographic characteristics were similar between the two groups (Table 1). Notably, there was no difference between the two groups for Canadian Cardiovascular Society and New York Heart Association classification, coronary artery disease status, left ventricular function and aortic transvalvular pressure gradients pre-TAVI. There was no statistically significant difference between the groups for the primary endpoint (4% vs. 4%; P = 1) (Fig. 1). Cardiac tamponade (4% vs. 4%; P = 1), readmissions (7% vs. 9%; P = 0.6) and all-cause mortality (1% vs. 1%; P = 1) at 30 days were low and equivalent in the two groups. Length of stay was similar with a median of 1-day post-TAVI in both groups. Cardiac readmissions (2% vs. 1%; P = 0.56), new permanent pacemaker implantation (5% vs. 4%; P = 0.73), cerebrovascular accident or transient ischaemic attack (0 vs. 1%; P = 0.32) at 30 days were similar between the two groups. There was no more than mild paravalvular leak in any of the cases with none in 63%, trivial in 29% and mild in 8%. The post-TAVI measurements and endpoints rates are presented in Table 2. There were no significant differences in echocardiographic parameters between the groups. To determine whether the intraprocedural echo was a valid surrogate of the routine next day echo we compared data from the intra-procedural ECHO with the routine next day ECHO in the group having both studies. This analysis revealed there were small differences between intraprocedural and next-day ECHO measurements (Table 3). The transvalvular parameters showed a tendency towards higher velocity (Vmax), mean gradients and larger indexed effective orifice area in the next-day versus the same-day post-TAVI echo. We speculate that a degree of left ventricular stunning, due to burst pacing during the procedure, resulted in lower values immediately post TAVI and that reversal of stunning by the following morning resulted in higher values on the next-day echo. However, importantly there were no instances of severe patient-prosthesis mismatch (VARC 3 definition) or change in paravalvular leak quantification between the 2 studies in any of the cases. For this analysis, the Valve Academic Research Consortium 3 definition was used with indexed effective orifice area ≤ 0.85 cm^2^/m^2^ in patients with body mass index (BMI)˂ 30 kg/m^2^ or ≤ 0.70 cm^2^/m^2^ in patients with BMI ≥ 30 kg/m^2^. Importantly, comparison of the LVOT dimensions in the cohort that had both studies revealed that there was no significant difference in the LVOT diameter between the post-TAVI in-cath lab study and next-day study.

**Table 1:** Baseline demographic characteristics

	Pooled (N = 200)	Same day (N = 100)	Next day (N = 100)	P
Gender male, n (%)	114 (57)	58 (58)	56 (56)	0.77
Age (years)	82 (77–86)	82 (77–85)	82 (77–86)	0.85
BMI (kg/m2)	27.4 (24.4–31.4)	27 (24.5–31.1)	27.9 (24.4–32.4)	0.64
BSA (m2)	1.87 (1.7–2.03)	1.88 (1.68–2.03)	1.87 (1.73–2)	0.91
Logistic Euroscore	10.4 (7–16)	10.7 (7.1–15.5)	10.2 (7–16.4)	0.76
DM, n (%)	53 (26.5)	27 (27)	26 (26)	0.87
Smoking, n (%)	110 (55)	53 (53)	57 (57)	0.57
Creatinine (umol/L)	88 (71–104)	84.5 (68.5–102)	89.5 (76–108)	0.24
Dialysis, n (%)	3 (1.5)	1 (1)	2 (2)	0.56
Previous MI, n (%)	29 (14.5)	10 (10)	19 (19)	0.07
Previous cardiac surgery, n (%)	25 (12.5)	11 (11)	14 (14)	0.52
Previous PCI, n (%)	36 (18)	13 (13)	23 (23)	0.07
CCS				
I-II, n (%)	32 (16)	14 (14)	18 (18)	
III-IV, n (%)	1 (0.5)	1 (1)	0	0.5
NYHA				
I-II, n (%)	48 (24)	25 (25)	23 (23)	
III-IV, n (%)	152 (76)	75 (75)	77 (77)	0.74
Vmax (m/sec)	4.2 (3.9–4.6)	4.2 (3.9–4.6)	4.2 (3.9–4.7)	0.45
mPG (mmHg)	43 (37–53)	43 (35–53)	44 (37–54)	0.35
pPG (mmHg)	70 (61–86)	70 (60–86)	70 (62–87)	0.41
AVA (cm^2^)	0.69 (0.56–0.80)	0.7 (0.6–0.8)	0.67 (0.51–0.78)	0.02
Pathology				
Stenosis, n (%)	196 (98)	99 (99)	97 (97)	
Mixed, n (%)	4 (2)	1 (1)	3 (3)	0.3
Bicuspid aortic valve, n (%)	20 (10)	12 (12)	8 (8)_	0.35
LVF				
> 50%, n (%)	171 (85.5)	85 (85)	86 (86)	
30–50%, n (%)	21 (10.5)	11 (11)	10 (10)	
< 30%, n (%)	8 (4)	4 (4)	4 (4)	0.97
CAD				
No, n (%)	145 (72.5)	76 (76)	69 (69)	
1vd, n (%)	29 (14.5)	13 (13)	16 (16)	
2vd, n (%)	20 (10)	9 (9)	11 (11)	
3vd, n (%)	6 (3)	2 (2)	4 (4)	0.68
LMS disease				
> 50%, n (%)	5 (2.5)	3 (3)	2 (2)	0.65

Categorical variables are expressed with N and %. Continuous variables are expressed with median and 25th, 75th percentile

*AVA* aortic valve area, *BMI* body mass index, *BSA* body surface area, *CAD* coronary artery disease, *CCS* Canadian Cardiovascular Society, *DM* diabetes mellitus; *LMS* left main stem, *LVF* left ventricular function, *MI* myocardial infarction, *mPG* mean pressure gradient, *NYHA* New York Heart Association, *PCI* percutaneous coronary intervention, *pPG* peak pressure gradient, *vd* vessel disease, *Vmax* maximum velocity

**Table 2:** Procedural characteristics and outcomes

	Pooled (N = 200)	Same day (N = 100)	Next day (N = 100)	P
Duration (min)	70 (60–80)	65 (60–75)	72 (66–81)	< 0.005
TAVI in TAVI	0	0	0	1
TAVI in SAVR, n, (%)	13 (6.5)	5 (5)	8 (8)	0.30
THV size (mm)	26 (23–26)	26 (23–26)	26 (23–29)	0.43
Post mPG^*^ (mmHg)	8 (5–10)	6 (4–8)	6 (4–8)	0.97
Post pPG^*^ (mmHg)	15 (10–18)	12 (9–16)	12 (8–16)	0.58
Post AVA^*^ (cm^2^)	2 (1.7–2.3)	2 (1.8–2.3)	2.1 (1.8–2.5)	0.94
Tamponade (detected in cath lab), n (%)	6 (3)	3 (3)	3 (3)	1
Tamponade (delayed detection), n (%)	2 (1)	1 (1)	1 (1)	1
LoS (days)	1 (1–1)	1 (1–1)	1 (1–1)	0.49
Urgent ECHO (after exit from cath lab), n (%)	4 (2)	2 (2)	2 (2)	1
PVL				
No, n (%)	126 (63)	66 (66)	60 (60)	
Trivial, n (%)	58 (29)	29 (29)	29 (29)	
Mild, n (%)	16 (8)	5 (5)	11 (11)	0.28
30-day readmissions, n (%)	16 (8)	7 (7)	9 (9)	0.6
In-hospital mortality, n (%)	2 (1)	1 (1)	1 (1)	1
30-day mortality, n (%)	2 (1)	1 (1)	1 (1)	1
30-day CVA, n (%)	1 (0.5)	0	1 (1)	0.32
30-day cardiac readmissions, n (%)	3 (1.5)	2 (2)	1 (1)	0.56
30-day new PPM, n (%)	9 (4.5)	5 (5)	4 (4)	0.73

Categorical variables are expressed with N and %. Continuous variables are expressed with median and 25th, 75th percentile

*CVA* cerebrovascular accident, *LoS* length of stay, *Post AVA* post-TAVI aortic valve area; Post mPG, Post-TAVI mean pressure gradient; post pPG, post-TAVI peak pressure gradient; PPM, permanent pacemaker; PVL, paravalvular leak; SAVR, surgical aortic valve replacement; THV, transcatheter heart valve

* For direct comparison between the two cohorts only intraprocedural ECHO results were compared

**Table 3:** Comparison of intraprocedural and next-day ECHO results within the next-day ECHO cohort

	Intraprocedural ECHO (N = 100)	Next day ECHO (N = 100)	P
Vmax, m/sec	1.7 (1.4–2)	2.1 (1.9–2.2)	< 0.005
pPG, mmHg	12 (7.7–15.9)	17.1 (13.8–19.4)	< 0.005
mPG, mmHg	6 (4.3–8.2)	10 (7.7–11.4)	< 0.005
EOA, cm^2^	2.1 (1.8–2.5)	1.9 (1.6–2.2)	0.019
EOAi, cm^2^/m^2^	1.13 (0.93–1.34)	1 (0.82–1.21)	0.033
PPM, n, (%)			
Moderate	5 (5)	12 (12)	
Severe	0	0	0.076
SVi, mL/m^2^	38.9 (32.7–49.8)	39.4 (29.8–52.1)	0.65
PVL ≤ mild	100%	100%	1

Categorical variables are expressed with N and %. Continuous variables are expressed with median and 25th, 75th percentile

*EOA* effective orifice area, *EOAi* effective orifice area indexed, *LVOTd* left ventricular outflow tract diameter, *mPG* post-TAVI mean pressure gradient, *pPG* post-TAVI peak pressure gradient, *PPM* patient-prosthesis mismatch, *PVL* para-valvular leak; *SVi* stroke volume index, *Vmax* maximum velocity

## Discussion

In this retrospective, single-centre study comparing intraprocedural transthoracic ECHO only with intraprocedural plus routine next day transthoracic ECHO following minimalist transfemoral elective TAVI with the Sapien 3 Ultra THV we found the following: (i) Intraprocedural ECHO appears to be as safe as additional routine next-day ECHO with respect to in-hospital mortality, need for urgent unplanned echo and detection of new tamponade on leaving the cath lab, (ii) Intraprocedural ECHO reduced sonographer workload, by obviating a routine next day ECHO, thereby improving resource utilisation and allocation.

Whilst the comparison of ECHO parameters between the intra-procedural ECHO and routine next day showed mild differences in the group undergoing both studies, we believe the differences are clinically unimportant and do not alter the view that the intraprocedural ECHO is a valid baseline study for future comparison. The analysis revealed small changes between the immediate post-procedure and next-day ECHO parameters (Table 3). However, there were no new cases of severe patient prosthesis mismatch on the next day echo and there was no change in the degree of paravalvular leak detected. Although there were more cases of moderate PPM in the next day this difference did not reach statistical significance. The reasons behind this observation are unclear, however, the small sample size prevents further meaningful subgroup analysis. Additionally, the observed rate of cardiac tamponade at 3% with an additional 1% with delayed identification in each group is consistent with the reported rates in contemporary studies ranging from 1–4%. This could be explained by the relatively older, comorbid and sicker population included in our cohort. [13–16]

Since the inception and first-in-human utilization of TAVI there have been significant and continuous refinements and improvements in the technique, safety and overall outcomes [17]. Previous large-scale studies and registries have highlighted the safety of transfemoral TAVI in the whole spectrum of surgical-risk patients spanning low-risk to inoperable cases. In-hospital, short- and medium-term outcomes appear to be better compared to medical management and at least comparable to surgical aortic valve re-placement both for balloon-expandable and self-expanding THVs [18–20]. Throughout the years, and particularly in the last decade, there has been considerable growth of structural interventions and particularly TAVI procedures. The most recent British Cardiovascular Intervention Society (BCIS) audit reported that the number of TAVI procedures increased from 66 cases in 2007 to a remarkable 6,719 cases in 2020/2021 in UK. The same trend has been observed in the US with more than 276,000 TAVI cases having been performed be-tween 2007 and 2019 with a steady increase over the last decade according to data from the Society of Thoracic Surgeons-American College of Cardiology Transcatheter Valve Therapy Registry. On a worldwide basis TAVI volume is estimated to reach 300,000 procedures per year. [21, 22]

This enormous growth in volume has highlighted the need for efficient resource utilization without impacting safety and outcomes and will become increasingly important. In keeping with this objective, intraprocedural ECHO only following TAVI appears safe and allows a more efficient use of echocardiography services. This practice obviates the need for routine next-day ECHO while still capturing the key parameters required for a baseline study post TAVI.

## Limitations

This was a retrospective study and as such selection bias could not be excluded. In this cohort only balloon-expandable valves were implanted potentially limiting the applicability of the results to other valves. Additionally, the sample size is modest, and this may impact the generalizability of the results which may not be applicable to cases other than transfemoral TAVI with conscious sedation. The rate of events was low which may impact on the power of the study results.

## Conclusions

Intraprocedural transthoracic ECHO only following elective transfemoral TAVI under conscious sedation with balloon-expandable THVs appears a safe and resource-efficient practice and may facilitate early discharge after uncomplicated TAVI. It obviates the need for routine next day ECHO thus, reducing the pressure on ECHO departments potentially allowing for more effective resource utilization. Future studies including higher number of cases may help shed more light into this matter.

## Data Availability

Not applicable.

## Abbreviations

ECHO: Echocardiography
TAVI: Transcatheter aortic valve implantation
THV: Transcatheter heart valve
